# A Biomimetic Human Multi-Cellular In Vitro Model of the Blood–Brain Barrier

**DOI:** 10.3390/ijms26083592

**Published:** 2025-04-11

**Authors:** John Saliba, Jessica Saliba, Marwan El-Sabban, Rami Mhanna

**Affiliations:** 1 Department of Anatomy, Cell Biology and Physiological Sciences, Faculty of Medicine, American University of Beirut, Beirut 1107 2020, Lebanon; jes11@aub.edu.lb; 2 Biomedical Engineering Program, Maroun Semaan Faculty of Engineering and Architecture, American University of Beirut, Beirut 1107 2020, Lebanon; 3 Department of Public Health, Faculty of Health Sciences, University of Balamand, Beirut 1100, Lebanon; jessica.saliba@balamand.edu.lb; 4 Department of Biology, Faculty of Sciences, Lebanese University, Beirut 1533, Lebanon

**Keywords:** blood–brain barrier, brain diseases, primary human astrocytes, primary human endothelial cells

## Abstract

Current in vitro models fail to recapitulate specific physiological properties of the human blood–brain barrier (BBB); hence the need for a reliable platform to study central nervous system diseases and drug permeability. To mimic the normally tight blood–brain interface, primary human endothelial cells (HAECs) and primary human astrocytes (A) were grown in a confined space of the physical scaffold created by gelatin methacrylate (GelMA) hydrogel to allow optimal astrocyte–endothelial cell direct/indirect interaction. Evidence for a physiologically relevant BBB was established by assessing the expression of tight junction markers conferring the barrier function, and by measuring biophysical attributes using the trans-endothelial electrical resistance (TEER) and the Evans blue albumin (EBA) permeability assay. An HAEC+A three-dimensional (3D) co-culture was associated with 12-fold higher *claudin-5 (CLDN5)* and *cadherin-1* (*CDH1* or *Epithelial [E]-cadherin)* transcriptional levels than two-dimensional (2D) models. This model conferred the highest TEER (45 Ω·cm^2^) in 3D HAEC+A, which value was 30 Ω·cm^2^ in 2D (*p* < 0.01) and 25 Ω·cm^2^ in 3D HAEC cultures (*p* < 0.001). Functionally, in 3D HAEC+A co-cultures, higher TEER resulted in 10-fold and 7-fold lower EBA permeability at 120 min, in HAECs alone or in to 2D co-cultures (*p* < 0.01). The established human primary cell model has acquired features mimicking the human BBB in vitro, and is now poised to be tested for the permeability of the BBB to pharmacological agents, parasites, cells (such as brain-tropic cancer cell metastasis) and any mechanisms that might involve traversing the BBB.

## 1. Introduction

The brain is a highly vascularized organ estimated to have a capillary supply to every neuron and a total surface area of almost 20 m^2^ [1]. The exchange of water-soluble metabolites, nutrients and ions between the blood and the central nervous system (CNS) microenvironment is tightly regulated by the blood–brain barrier (BBB) [2]. The BBB is the most efficient barrier in the body, rendering it an obstacle to drug delivery to the brain. It is composed of specialized endothelial cells in addition to astrocyte foot processes and pericytes that line the brain capillaries and actively control the passage of cells and a variety of molecules, contributing to the BBB [3]. In fact, the BBB is impermeable to all large-molecule drugs and most small-molecule drugs, with less than 2% being allowed to cross [4]. This underscores the limited treatment strategies and ineffective systemic therapies targeting brain metastatic tumors, which are the most common tumors in adults associated with poor prognosis [5]. While the BBB is highly impermeable to drugs, conversely, it was shown to be corruptible by malignant cells metastasizing to the brain. Therefore, formulation strategies are being developed for CNS drugs to traverse the BBB using endogenous transporters found on the endothelium of the capillaries.

Studying cancer metastasis and drug delivery mechanisms through the BBB is commonly performed using both in vivo and in vitro models [6]. Several micro-physiological and three-dimensional (3D) microfluidic models have been developed [7,8,9,10], with the ultimate purpose of mimicking physiological and pathological processes in vitro [11]. The advantages of in vitro models compared to in vivo models are reduced costs, reproducibility, and high throughput, which have encouraged the development of novel in vitro BBB models. The capacities of in vitro models to control biological and environmental factors are vital in studying specific cell–cell interactions and responses to external stimuli. Most of current in vitro BBB models that study brain-tropic cell metastasis do not faithfully replicate the native BBB. These models rely on creating an endothelial monolayer on Matrigel™ or fibronectin-coated cell culture inserts incubated in astrocyte-conditioned media or with astrocytes and fibroblasts at the bottom of the wells [12,13,14]. Several bi-cellular models of the BBB have been developed, some using endothelial cells generated from induced pluripotent stem cells (iPSCs) and collagen from rat tails [15,16], others using astrocytes from murine origin [17], or immortalized endothelial cell lines, which modifies the cells’ morphology and may result in compromised barrier function [17,18,19,20]. In vitro BBB models provide great insight into brain vascular diseases and the administration of drugs to the brain, and should be designed to answer specific questions, within the constraints of resources, the specific need for high-throughput screening, the length of culture and endpoints, etc. [19]. BBB models are essential tools used to determine the potential therapeutic permeability of drugs delivered to the brain and the mechanisms involved in the cell biology of the tight junctions [21]. Importantly, the lab-on-a-chip technology is gaining terrain, and is currently approved by the United States Food and Drugs Administration for a variety of applications, including for evaluating drug toxicity [22]. However, most of the current models used to study cancer metastasis to the brain or to evaluate CNS-targeted drugs are a simplified representation of the BBB, do not use exclusive human cells, use iPSC-derived endothelial cells or a monocellular system, and do not recapitulate the 3D in vivo tissue complexity [16,23,24,25].

This manuscript describes the development of a novel biomimetic BBB model, employing exclusively primary human cells and easy-to-manipulate and inexpensive gelatin methacrylate (GelMA) hydrogel, for in vitro studies of CNS diseases and the movement of molecules and cells to the CNS. The BBB model was created by seeding primary human astrocytes and primary endothelial cells on a physical scaffold of GelMA hydrogel. This novel model allows communication between human primary astrocytes and endothelial cells, which enhances endothelial barrier function [20,26]. The model was characterized by assessing the physical properties of the GelMA hydrogel that served as a physical scaffold allowing endothelial cell/astrocyte interaction. The model was then used to investigate the barrier function of the 3D cell system by trans-endothelial electrical resistance (TEER) and Evans blue albumin (EBA) permeability assay. This novel biomimetic BBB model relies exclusively on human primary cells, and is intended for in vitro studies of drug delivery and cancer metastasis to the brain.

## 2. Results

### 2.1. Characterization of the GelMA Hydrogels

**Figure 1:**
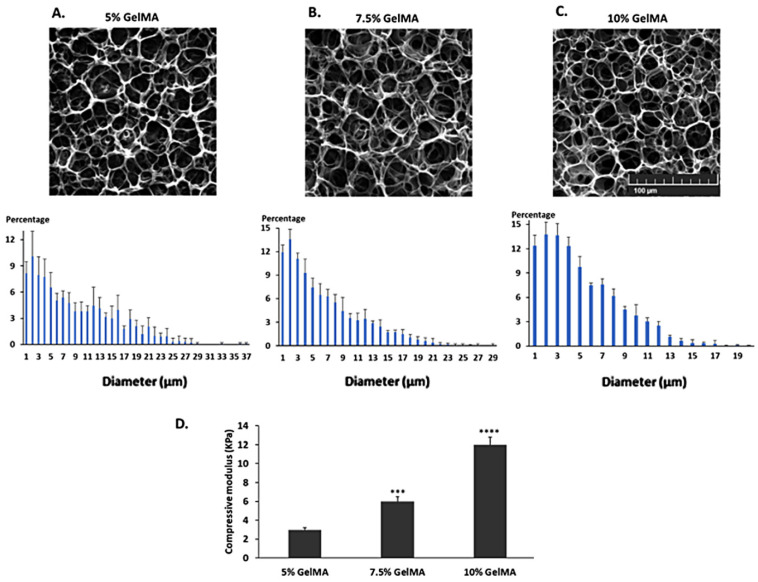
Characterization of the GelMA hydrogels. Representative scanning electron microscopy images of (**A**) 5% GelMA, (**B**) 7.5% GelMA and (**C**) 10% GelMA hydrogels. Lower panels display the distribution of pore sizes, after pore diameter was analyzed on the ImageJ software (version 1.54g). (**D**) Compressive moduli of 5%, 7.5% and 10% GelMA hydrogels. The data summarize results from at least 3 independent experiments. One-way ANOVA: *** *p* < 0.001 and **** *p* < 0.0001.

Scanning electron microscopy (SEM) images of the different concentrations of GelMA hydrogels were analyzed to determine the pore size range and distribution (Figure 1A–C). SEM images of the GelMA hydrogels revealed that most pore diameters ranged from 1 to 7 µm. The increase in concentration led to fewer larger pores. In particular, the 10% GelMA hydrogels had virtually no pores larger than 13 μm in diameter (Figure 1C). The mechanical properties of the GelMA hydrogels were also studied and the compressive Young’s moduli of the hydrogels were determined (Figure 1D). We found that 5% GelMA had the lowest Young’s modulus of 3.1 KPa, which is closest to the Young’s modulus of the native brain (~0.9 KPa) [27].

### 2.2. Human Astrocytes Cultured in GelMA Hydrogels Are Viable

**Figure 2:**
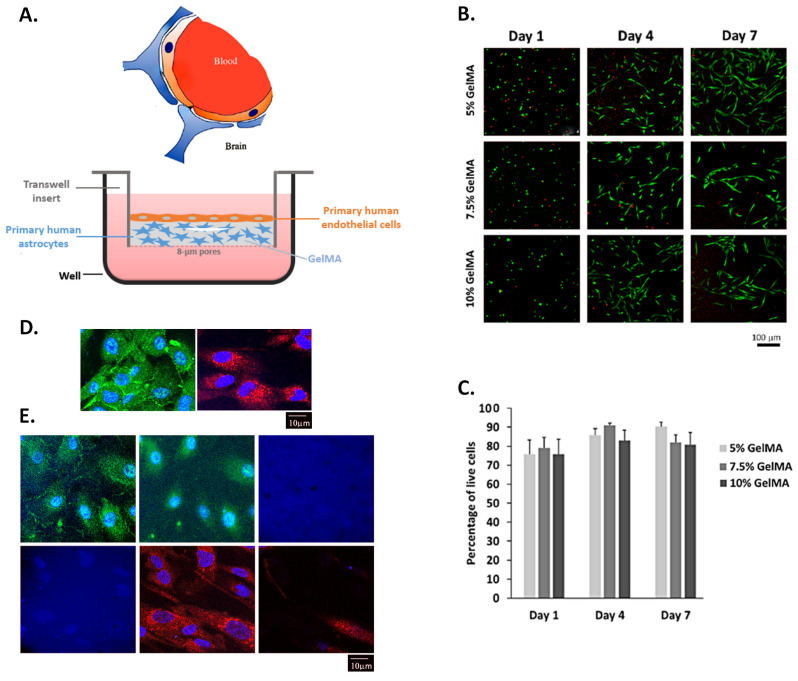
Viability of primary human astrocytes grown in a physical scaffold made of GelMA hydrogel. (**A**) Inserts with microporous membranes were used to plate the astrocytes in GelMA scaffold. HAECs were seeded on top of the 45 μm-thick GelMA hydrogel after astrocytes had proliferated in culture. (**B**) Fluorescent micrographs of astrocytes using Live/Dead assay Calcein AM (green) and Propidium Iodide (red) on days 1, 4 and 7. (**C**) Viability of astrocytes grown in different concentrations of GelMA hydrogel. Percentage of live cells out of the total cell number calculated from live/dead images (mean ± standard error of the mean from 3 independent experiments). (**D**) Fluorescent micrographs of HAECs (evidenced by membranous ZO-1 staining, Alexa Fluor 488) and astrocytes (evidenced by cytoplasmic GFAP staining, Texas Red) seeded on a GelMA scaffold. Nuclei are stained with DAPI (blue). (**E**) Optical sections, 3 μm-thick, of ZO-1-stained HAECs and GFAP-positive astrocytes, across the height of the insert. The top left panel is the top of the insert, followed by images taken at 3 μm intervals, until the bottom right panel (bottom of the insert). Micrographs are representative of at least 2 experiments designed for fluorescence imaging of the proposed BBB model.

The viability of primary human astrocytes cultured as shown in Figure 2A was assessed using the LIVE/DEAD™ Cell Viability/Cytotoxicity Kit, according to the manufacturer’s instructions. Figure 2B shows live cells fluorescing in green and dead cell nuclei stained red, in 5%, 7.5% and 10% GelMA hydrogels on days 1, 4 and 7 post-seeding. In particular, astrocytes have increased in number and extended their processes into the GelMA hydrogel. Figure 2C shows the percentage of live cells, counted from different fields of the fluorescent micrographs. We observed a high viability (~80%) across the different concentrations of GelMA hydrogels (Figure 2C). The highest viability was observed with the 5% GelMA hydrogels with greater than 90% viability after 7 days in culture. For the remainder of the study, the 5% GelMA hydrogel was used to study the growth of primary human aortic endothelial cells (HAECs) with the primary human astrocytes and the establishment of a functional barrier. Figure 2D depicts zonula occludens (ZO)-1-stained HAECs and glial fibrillary acidic protein (GFAP)-positive primary human astrocytes seeded in 5% GelMA hydrogel, and Figure 2E is a representative optical sectioning (3 µm-thick z-stacks) of an HAEC–astrocyte-seeded insert, across the height of the insert, showing HAECs and astrocytes at different levels of the GelMA hydrogel (evocative of Figure 2A).

### 2.3. Tight and Adherens Junction Proteins Are Expressed in This Model of the BBB

**Figure 3:**
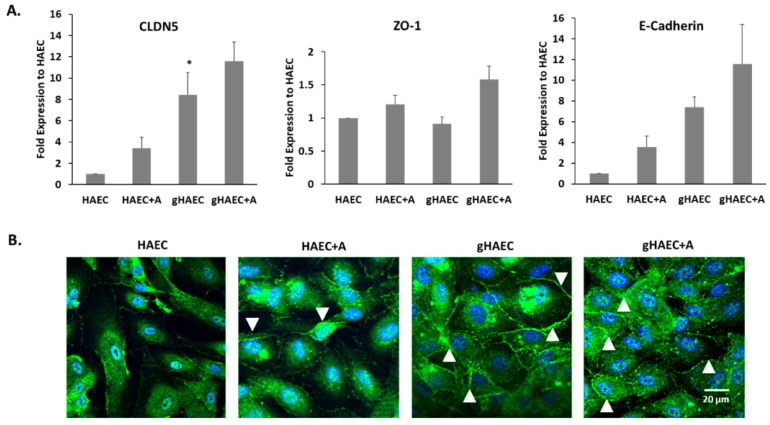
Expression of tight junction proteins by HAECs grown on GelMA hydrogels with or without astrocytes. (**A**) Normalized gene expression of tight junction markers *CLDN5* and *ZO-1* and adherens junction marker *E-cadherin* by HAECs. (**B**) Immunofluorescence micrographs of *ZO-1* expressed by HAECs (green). Nuclei are stained with DAPI (blue). White arrowheads indicate points of cell-to-cell contact. HAEC: HAECs cultured on inserts. HAEC+A: co-culture of HAECs and astrocytes on inserts. gHAEC: HAECs cultured on GelMA. gHAEC+A: co-culture of HAECs on top of astrocytes in a scaffold made of GelMA. Data summarize results obtained from at least 3 independent experiments, run in duplicate (for qRT-PCR). One-way ANOVA * *p* < 0.05.

The expressions of tight junction genes, characteristic of the BBB, were evaluated in HAECs grown on cell culture inserts, with and without astrocytes, in HAECs grown on 5% GelMA and in HAECs grown on 5% GelMA with astrocytes. The expression of *CLDN5*, *ZO-1* and *E-cadherin* was highest in the latter cell model (Figure 3A). The expression of *CLDN5* was significantly higher when cells were grown in 3D (i.e., using GelMA hydrogel); *CLDN5* transcriptional levels were 9-fold higher in the 3D gHAEC set than in the 2D HAEC set (*p* < 0.05), and 12-fold higher in the 3D gHAEC + astrocyte set than in the 2D HAEC + astrocyte set. Figure 3B shows representative micrographs of ZO-1 tight junction proteins expressed in HAECs, whether alone or with astrocytes.

### 2.4. Efficiency of the Barrier Function

**Figure 4:**
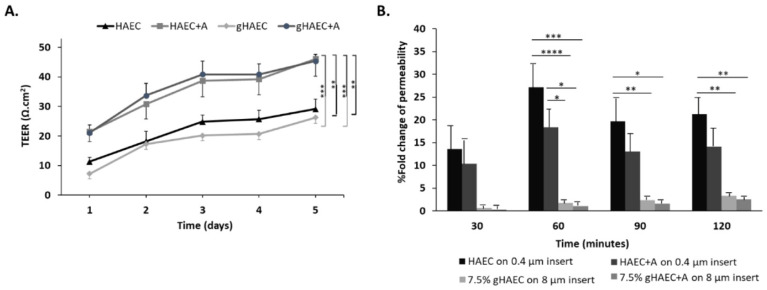
Assessment of the barrier function of the developed BBB models. (**A**) TEER of HAEC measured for 5 days. (**B**) EBA permeability assay. At 120 min, all conditions are compared to gHAEC+A, and change of permeability is reported as a function of insert pore sizes. Error bars represent the standard error of the mean from at least 4 independent experiments. Two-way ANOVA: * *p* < 0.05, ** *p* < 0.01, *** *p* < 0.001, **** *p* < 0.0001. HAEC: HAECs cultured on 0.4 μm-pore inserts. HAEC+A: co-culture of HAECs and astrocytes on 0.4 μm-pore inserts. gHAEC: HAECs cultured on GelMA in 8 μm-pore inserts. gHAEC+A: co-culture of HAECs on top of astrocytes in a scaffold made of GelMA on 8 μm-pore inserts.

To study the functionality of tight junctions contributed by HAECs, TEER measurements of all four conditions were reported over 5 days (Figure 4). The TEER measurements showed that the GelMA models had the highest TEER. Specifically, Figure 4A shows the highest TEER in the gHAEC + astrocyte set compared to 2D HAECs (*p* < 0.01) or to 3D gHAECs (*p* < 0.001). The co-culture of HAECs and astrocytes conferred the highest TEER, whether in 2D or in 3D settings. In particular, after 5 days of co-culture, TEER measurements for HAEC + astrocyte conditions averaged at 45 Ω·cm^2^, whether in 2D co-cultures in inserts or with GelMA hydrogels, which is significantly higher than HAECs alone in 2D (TEER = 30 Ω·cm^2^, *p* < 0.01) or 3D cultures (TEER = 25 Ω·cm^2^, *p* < 0.001).

Similarly, 3D HAECs or 3D HAECs + astrocytes (conditions using GelMA) allowed for the least permeability of EBA, compared to 2D HAECs or 2D HAECs + astrocytes (Figure 4B). At 120 min, gHAEC + astrocytes allowed for a 10-fold lower permeability than HAEC in 2D (*p* < 0.01) and for a 7-fold lower permeability than HAEC + astrocytes in 2D (*p* < 0.01). At the earlier time point of 60 min, the differential permeability between 2D endothelial cell cultures and 3D gHAEC + astrocyte models was even more pronounced (*p* < 0.0001).

Collectively, although HAECs alone do express tight junction proteins, especially in 3D culture, the presence of astrocytes is essential for a functional barrier.

## 3. Discussion

There have been numerous attempts to engineer the human BBB in in vitro 3D models to study the delivery of CNS drugs and other mechanisms occurring across the BBB, ranging from pathogen and foreign cell guarding to allowing the delivery of nutrients to the CNS tissue [28,29,30]. Some of these models recapitulate, with varying veracity, certain aspects of the BBB, notably the presence of more than one cellular component in the neurovascular unit, the effect of sheer flow, the 3D structure, as well as the formation of the distinctive tight junctions between endothelial cells [31,32].

Microfluidic platforms are commonly made of a biomaterial on and inside which tissue-specific cells are grown, and fluid flow (shear or interstitial flow) and electrochemical stimuli are applied [30]. Microfluidic platforms are made of one of numerous naturally derived (Chitosan, Matrigel) or synthetic biomaterials (Poly-(ε-caprolactone), Poly-(dimethyl-siloxane)), molded into the desired scaffold architecture [33,34]. While microfluidic devices enable the precise control of fluid flow and some have an optical transparency making them suitable for imaging [35], they do not readily allow exchange across the duct walls, unlike normal tissues in vivo. Therefore, the study of drug delivery and cell permeability might be difficult. Some works are attempting to recapitulate cell and substance transport across the microfluidic system walls, but the applicability of these models remains limited [36,37].

Among available biomaterials, one that has gained popularity in recent years is GelMA hydrogel, a synthetic material characterized by biocompatibility and mechanical tenability [38,39], in addition to the possibility of incorporating various biomaterials to improve its physical and chemical properties, scaffold architecture, conductivity and porosity, and create a hospitable growth environment for a wide variety of cells [33,39].

This study aimed at creating an in vitro BBB model, using GelMA as a biocompatible scaffold as well as primary human astrocytes and primary human endothelial cells (HAECs), to test tight junction formation and permeability across the BBB. Very few studies use exclusively primary human endothelial cells and primary human astrocytes to reproduce the BBB in vitro. In fact, iPSCs have been used to differentiate into endothelial cells [15,16,40,41,42,43,44], which needs to be proven as a good surrogate for normal vascular endothelial cells, and mouse-derived neural cells or astrocytes are still used in lieu of human cells [17,45]. While models employing more than the two main cell types involved in the BBB (namely, endothelial cells and astrocytes) exist, the relative simplicity of even monocellular (endothelial cell-based) models endorses their use for toxicity, proliferation, and transport studies, and the characterization of immune cells’ secretions [23,46]. Our model utilizes a bi-cellular design exclusively composed of primary human cells, a design superior to monocellular models [24,25,47,48], and to models utilizing cells of non-human origin, immortalized cell lines or fetal astrocytes that were found to be non-dividing in culture [17,18,25]. A recent study, published in 2025, uses a commercially available 3D model, known as the Real Architecture for Tissue (RAFT™) 3D co-culture system, which was seeded with all three cell types constituting the BBB (i.e., astrocytes, endothelial cells and pericytes), in addition to a cell line of special interest in Parkinson’s disease [24]. This model might be superior to bicellular models, but is expensive [24] if meant to be used for extensive drug screening purposes, or assessments of mechanisms of brain-tropic cell metastasis, or parasite invasion of the brain microenvironment. Moreover, the RAFT model resulted in a decreased cell size and inability to detect some tight junction proteins, and still does not emulate the blood flow observed in vivo [24]. Additionally, the Transwell system used in the present model offers flexibility, versatility, the possibility of physical, paracrine and direct contact and communication with the cell types involved, ease of performance of the TEER and functional assays, etc., which benefits are further improved by the addition of hydrogels that facilitate cell–cell interaction [49].

For a better understanding of the conditions promoting the barrier function conferred by the interaction of the primary human cells seeded together in the GelMA hydrogel, the pore size distribution inside the hydrogel (revolving around 3 μm in the 5% hydrogel) was reported and compared to the dimensions of endothelial cells and protoplasmic human astrocytes, to ensure their ability to infiltrate the GelMA hydrogel. Endothelial cells measure roughly 50–70 μm in length, 10–30 μm in width and 0.1–10 μm in thickness [50]. The cell body of protoplasmic astrocytes ranges from 10 to 20 μm and their processes radiate out for a maximal average length of 97.9 ± 5.2 μm, and the diameter of the thickest processes is about 2.9 ± 0.18 μm [51]. The astrocytes’ cell body can infiltrate larger pores inside the GelMA hydrogel and astrocytic processes, being around 3 μm thick, and can weave its way inside the pores. In fact, seeding astrocytes and endothelial cells on either sides of a Transwell membrane with 3 μm-wide pores was shown to improve connections between the two cell types [52]. The Young’s modulus (the elasticity of the material or its ability to undergo a change in length under a tensile or compressive stress [33]) was compared across different concentrations of the GelMA hydrogel (5%, 7.5% and 10%). The concentration that yielded a Young’s modulus closest to that of brain tissue (~0.9 KPa) [27] and allowed for optimal cell adherence and growth (i.e., 5% GelMA) was adopted throughout this study. Other studies have reported the ability of endothelial cells to form capillary networks inside acellular 3D scaffolds of various stiffnesses and elasticities, and found that a higher elastic modulus resulted in endothelial cells remaining as single cells and failing to form networks [53,54,55].

This study verified the adherence of the primary human astrocytes to the GelMA scaffold, and their viability. The GelMA scaffold was intended to position the astrocytes in close proximity to the basolateral aspect of the endothelial cells, in an effort to enhance and favor direct and paracrine interaction between the two cell types. It is not meant to substitute the parenchyma of the brain, which is composed of complex and diverse extracellular matrix proteins, depending on anatomical location. We anticipate that this 3D model can be used to introduce additional cells encountered inside and in the vicinity of the brain tissue, contributing to the efficiency of the BBB, such as pericytes and immune cells [56,57]. A recent meta-analysis comparing and contrasting in vitro models of the BBB in diseases of the brain (glioblastoma, Parkinson’s disease and others) recommended a higher ratio of astrocytes (or pericytes) to endothelial cells [58]. Although the cell densities used in this model may not match recommendations from the meta-analysis [58], this will be taken into consideration in future experiments.

The configuration obtained in our model has resulted in the expression of tight junction markers by endothelial cells, *ZO-1* and *CLDN5*, as well as the adherens junction marker *CDH1* or *E-cadherin*, in accordance with the literature [20,44]. The functionality of these tight junctions was evaluated by reporting the electrical impedance of endothelial cell monolayers and by an EBA permeability assay. TEER measurements were the highest in co-culture settings combining endothelial cells and astrocytes. This contradicts with a comparative study on four immortalized human brain capillary endothelial cell lines with/without co-culture with immortalized human astrocytes on cell culture inserts, in terms of barrier tightness and paracellular permeability [20]. The study found that the co-culture of endothelial cells with astrocytes did not result in a TEER increase [20]. A BBB model in rats reported the highest TEER of 400 Ω·cm^2^ in a triple co-culture system of endothelial cells, pericytes, and astrocytes [59]. Another triple co-culture protocol intended to mimic the BBB using human cells exclusively has been proposed [60]. The system used endothelial cells derived from cluster of differentiation 34 (CD34)-positive hematopoietic stem cells isolated from human umbilical cord blood, and has cells seeded on either side of cell culture inserts. Despite its usefulness in studying the permeability of an in vitro BBB model, it almost exclusively relies on tight and adherens junctions and does not reproduce the complexity of the 3D architecture of the BBB [60]. A recent study by our group used polyethylene glycol (PEG) hydrogels modified with biomimetic peptides as a 3D structure to populate with human astrocytes and the endothelium cell of vessel 304 (ECV-304) cell line, achieving a TEER of 55 Ω·cm^2^ [61]. The current model uses a simpler, less manipulated 3D scaffold, which enhances cell–cell interaction across the Transwell membrane and exclusive primary human cells [49]. The EBA assay showed that the co-culture of endothelial cells on top of astrocytes in GelMA hydrogel resulted in the significantly lower permeability of albumin, a bio-pharmaceutically relevant molecule that does not cross a healthy BBB, compared to endothelial cells alone or the co-cultured cells in 2D, and therefore prevented BBB breach by paracellular transport [37,62,63].

Collectively, the results from this study show that a simple and relatively inexpensive model of the 3D (GelMA) co-culturing of primary human astrocytes with primary human endothelial cells can promote the expression and activity of tight and adherens junction proteins, and establish a BBB with demonstrated increased TEER and decreased permeability. The GelMA hydrogel can be further modulated with adjusted photo-initiator concentration and the use of dental curing light instead of UV for polymerization and the formation of larger pores, as well as with the addition of natural and synthetic biomaterials for a wider range of applications and cell populations, and incorporation into a more advanced microfluidic brain-on-a-chip model [39,64]. The strength of this proposed model lies in the use of physiologically pertinent human primary cells that maintain a barrier function on par with other published works, and is amenable to modulation by further enhancing cellular and structural components. A more refined platform will prove valuable for the screening and delivery of drugs to the brain, and for studying brain-tropic parasites and cancer cell metastasis.

## 4. Materials and Methods

### 4.1. GelMA Synthesis

GelMA was prepared as previously described [65]. Briefly, type A gelatine (cat# G2500, Sigma-Aldrich, St Louis, MO, USA) produced from porcine skin was dissolved at 10% (*w*/*v*) in 0.1 M sodium carbonate–bicarbonate buffer (3.18 g sodium carbonate and 5.86 g sodium bicarbonate) at 50 °C, while stirring for 30 min. Methacrylic anhydride (cat# 276685, Sigma-Aldrich, St Louis, MO, USA) was added at a ratio of 0.1 mL:1 g of gelatine at 50 °C. Methacrylic anhydride was added dropwise every 30 min for 3 h to the gelatine solution with pH adjustment to 9. After 3 h of reaction, the solutions were readjusted to a pH of 7.4, and diluted with phosphate-buffered saline (PBS). The diluted solution was then dialyzed for a week in distilled water. The solution was then filtered and stored at −80 °C overnight followed by lyophilization for a week. The obtained foam was stored at −20 °C. the GelMA prepolymer solution was prepared by dissolving GelMA in 0.5% (*w*/*v*) photo-initiator solution of 2-Hydroxy-4′-(2-hydroxyethoxy)-2-methylpropiophenone (referred to as Irgacure 2959, cat# 410896, Sigma-Aldrich, St Louis, MO, USA).

### 4.2. Scanning Electron Microscopy

GelMA hydrogels of different concentrations (5%, 7.5% and 10%) were prepared to analyze their internal structure. The hydrogels were hydrated with PBS and then frozen at −80 °C overnight. The frozen hydrogels were then lyophilized for 3 h and sputter-coated with 5 nm of platinum. The surface topography was imaged with the scanning electron microscope (MIRA3, TESCAN, Dortmund, Germany), available at the Kamal A. Shair Central Research Science Laboratory at the American University of Beirut, Lebanon.

### 4.3. Cell Culture

HAECs (cat# CC-2535, Lonza, Vacaville, CA, USA) and primary human astrocytes (cat# 10HU-035, iXCells Biotechnologies, San Diego, CA, USA) were cultured in endothelial cell growth medium and astrocyte growth medium (cat# MD-0039, iXCells Biotechnologies, San Diego, CA, USA), respectively, and were maintained in an incubator at 37 °C with 5% CO_2_. HAECs were initially thawed and cultured in 75 cm^2^ flasks and the human astrocytes were thawed and cultured in 75 cm^2^ flasks coated with 0.01% poly-L-lysine (PLL), as per the vendor’s protocol.

For barrier formation, HAECs and human astrocytes were cultured on 12-well Transwell™ inserts (cat# 353180, Corning, Amsterdam, The Netherlands). Primary human astrocytes were seeded on the abluminal side of 0.4 µm-pore-size inserts coated with PLL at a density of 2 × 10^4^ cells per insert and cultured for 4 days. The HAECs were cultured on the luminal side of the inserts at a density of 3 × 10^4^ cells per insert and grown for 5 days. For 3D co-culturing, human astrocytes were mixed with 40.5 μL GelMA at a density of 10^6^ cells/mL. The cell-laden GelMA solution was then placed in the luminal side of 8 µm-pore size inserts to obtain a homogeneous layer and cross-linked with UV to induce its polymerization. After 4 days, 2 × 10^4^ HAECs were seeded on top of the 45 μm-thick hydrogel. All culture models were incubated at 37 °C with 5% CO_2_. Cell densities were determined in this study based on viability assays and the presumption that the astrocytes will increase in number over the 5-day culture. This is comparable to other models [66].

### 4.4. Assessment of Cell Viability

The viability of human astrocytes grown in different concentrations of GelMA hydrogels was assessed using LIVE/DEAD™ Viability/Cytotoxicity Kit for mammalian cells (cat# L3224, Invitrogen, Eugene, OR, USA), per the manufacturer’s protocol. Briefly, the live/dead solution was added to the hydrogel and incubated for 30 min before fluorescence imaging using a laser-scanning confocal microscope (LSM710, Zeiss, Jena, Germany), operated by the Zen™ software (Zen black 3.0 SR, Zeiss, Jena, Germany). The results are displayed as representative fluorescent micrographs and as the percentage of live cells to the total cell number calculated from live/dead images (mean ± standard error of the mean).

### 4.5. Assessment of Gene Expression

Total RNA was extracted using the RNeasy^®^ Plus mini kit (cat# 74134, Qiagen, Hilden, Germany) as per the manufacturer’s instructions, and quantitative reverse transcription real-time polymerase chain reaction (qRT-PCR) was performed, as previously reported [67]. Forward (F) and Reverse (R) 5′3′ primer sequences for the three genes of interest were as follows: *claudin 5* (*CLDN5)* F—CTGGACCACAACATCGTGA and R—CACCGAGTCGTACACTTTGC; *glyceraldehyde-3-phosphate dehydrogenase* (*GAPDH*) F—TGGTGCTCAGTGTAGCCCAG and R—GGACCTGACCTGCCGTCTAG; *cadherin 1* (*CHD1)* or *epithelial (E)-cadherin* F—GTGACTGATGCTGATGCCCCCAATACC and R—GACGCAGAATCAGAATTAGGAAAGCAAG; *tight junction protein 1* (*TJP1* or *ZO-1*) F—CAGCCGGTCACGATCTCCT and R—TCCGGAGACTGCCATTGC. All experiments were carried out in duplicates and independently performed at least three times.

### 4.6. Expression and Localization of ZO-1 and GFAP by Immunofluorescence

Cells were grown on inserts and in GelMA hydrogels that served as physical scaffold to permit cell–cell interaction. After HAECs had formed a tight monolayer and human astrocytes had grown confluent, cell preparations were washed with PBS and fixed in ice-cold methanol at −20 °C overnight. After washing with PBS wash, non-specific binding was blocked by incubating the cells with 3% normal goat serum and bovine serum albumin (BSA) solution in PBS for 1 h at room temperature. The inserts were then cut, placed in a humidified chamber and incubated with primary antibodies at 4 °C overnight. Primary antibodies against the tight junction protein ZO-1 (rabbit anti-ZO-1, cat# 61-7300, Invitrogen, Carlsbad, CA, USA) and against astrocyte intermediate filament GFAP (mouse anti-GFAP, cat# ab4648, Abcam, Waltham, MA, USA) were added. The cells were then washed three times with PBS + 0.05% Tween and the secondary antibody was added—goat anti-rabbit Alexa Fluor 488 and goat anti-mouse Texas Red—at room temperature for 1 h. The cells were then washed three times with PBS + 0.05% Tween and counterstained with Hoechst 33342 at room temperature for 10 min. After washing with PBS two times, cell preparations were mounted on microscope slides using ProLong Antifade (ProLong Diamond Antifade Mountant, cat# P36961, Molecular Probes, Carlsbad, CA, USA). Immunofluorescence micrographs were observed and single-plane and optical section images were acquired using the LSM710 microscope operated by the Zen software.

### 4.7. Measurement of TEER

TEER values were acquired daily after the seeding of cells, on days 1 to 5, using an epithelial volt/ohm meter (EVOM2, World Precision Instruments, Sarasota, FL, USA) with STX2 chopstick electrodes, as previously described [61]. The media of the inserts were changed daily and left to equilibrate for 30 min before measurements. TEER values (acquired in duplicates from 4 independent experiments) were corrected by subtracting the values of inserts without cells, and multiplied by the effective membrane surface area of the inserts to obtain the unit area resistance (Ω·cm^2^).

### 4.8. Measurement of Permeability

Barrier permeability was analyzed using EBA assay (cat# E2129, Sigma-Aldrich, St. Louis, MO, USA). The BBB models were washed with PBS and a permeability buffer (141 mM NaCl, 2.8 mM CaCl_2_, 1 mM MgSO_4_, 4 mM KCl, 1 mM NaH_2_PO_4_, 10 mM glucose and 10 mM HEPES, pH 7.4) was added to the abluminal side, while Evans blue dye (340 μg/mL) and BSA (10 mg/mL) were added to the luminal side (Evans blue is a dye that binds albumin [EBA] [68]). The cells were incubated at 37 °C for 30 min, and the levels of Evans blue dye in the abluminal side were measured using a spectrophotometer with an excitation/emission wavelength ratio of 540/680 nm for EBA. EBA concentrations were determined using a standard curve. Data are reported as a function of insert pore sizes (0.4 μm and 8 μm pore size).

### 4.9. Statistical Analysis

Quantitative data were reported as averages ± standard deviation or standard error of the mean. The *p* value was determined and considered significant at 0.05. Differences between experimental groups were assessed using Student *t*-test (when comparing averages between experimental and control sets), one-way analysis of variance (ANOVA) or by two-way ANOVA followed by Tukey’s multiple comparisons test (for analyses between experimental groups). All experiments were performed with at least 3 independents repeats, unless specified otherwise.

## 5. Conclusions

This study describes and validates the establishment of an in vitro model of the BBB that circumvents the shortcomings of other currently used models described in the literature, i.e., the 2D architecture, the use of non-human or iPSCs to populate the biomaterial scaffold, and the use of a single cell type. While the experimental concept and design adopted in this study are not novel, the exclusive use of primary human endothelial cells and primary human astrocytes to reproduce the BBB in vitro is unique. Although the testing of the model was performed under static conditions, we are currently devising a microfluidic system to allow for the control of fluid flow along the endothelial layer. In conclusion, the strength of this work resides in the exclusive use of primary human cells, in the use of a biocompatible, biodegradable and modular 3D scaffold (GelMA hydrogel), and in the relative ease of reproducing it for in vitro experiments. This model warrants further investigation to test drug permeability and the migration of cancer cells in and out of the CNS.

## Data Availability

Raw data are available upon reasonable request.

## Abbreviations

A: astrocytes. BBB: blood–brain barrier. BSA: bovine serum albumin. CNS: central nervous system. EBA: Evans blue albumin. HAEC: human aortic endothelial cells. GelMA: gelatine methacrylate. PLL: poly-L-lysine. SEM: Scanning electron microscopy. TEER: trans-endothelial electrical resistance. *ZO-1*: zonula occludens. *CLDN5*: claudin-5.
